# Working With Parents to Prevent Childhood Obesity: Protocol for a Primary Care-Based eHealth Study

**DOI:** 10.2196/resprot.4147

**Published:** 2015-03-25

**Authors:** Jillian LS Avis, Andrew L Cave, Stephanie Donaldson, Carol Ellendt, Nicholas L Holt, Susan Jelinski, Patricia Martz, Katerina Maximova, Raj Padwal, T Cameron Wild, Geoff DC Ball

**Affiliations:** ^1^Department of PediatricsFaculty of Medicine & DentistryUniversity of AlbertaEdmonton, ABCanada; ^2^Department of Family MedicineFaculty of Medicine & DentistryUniversity of AlbertaEdmonton, ABCanada; ^3^Primary Care & Chronic Disease ManagementAlberta Health ServicesEdmonton, ABCanada; ^4^Primary Health CareAlberta Health ServicesEdmonton, ABCanada; ^5^Faculty of Physical Education & RecreationUniversity of AlbertaEdmonton, ABCanada; ^6^Chronic Disease Management ResearchAlberta Health ServicesCalgary, ABCanada; ^7^Public Health and Wellness BranchHealth Services DivisionMinistry of Health, Government of AlbertaEdmonton, ABCanada; ^8^School of Public HealthUniversity of AlbertaEdmonton, ABCanada; ^9^Department of MedicineFaculty of Medicine & DentistryUniversity of AlbertaEdmonton, ABCanada; ^10^Pediatric Centre for Weight and HealthStollery Children's HospitalEdmonton, ABCanada

**Keywords:** body weight, Canada, child, childhood obesity, Internet, parents, prevention, primary health care

## Abstract

**Background:**

Parents play a central role in preventing childhood obesity. There is a need for innovative, scalable, and evidence-based interventions designed to enhance parents’ motivation to support and sustain healthy lifestyle behaviors in their children, which can facilitate obesity prevention.

**Objective:**

(1) Develop an online screening, brief intervention, and referral to treatment (SBIRT) eHealth tool to enhance parents’ concern for, and motivation to, support children’s healthy lifestyle behaviors, (2) refine the SBIRT eHealth tool by assessing end-user acceptability, satisfaction, and usability through focus groups, and (3) determine feasibility and preliminary effectiveness of the refined SBIRT eHealth tool through a randomized controlled trial.

**Methods:**

This is a three-phase, multi-method study that includes SBIRT eHealth tool development (Phase I), refinement (Phase II), and testing (Phase III). Phase I: Theoretical underpinnings of the SBIRT tool, entitled the Resource Information Program for Parents on Lifestyle and Education (RIPPLE), will be informed by concepts applied within existing interventions, and content will be based on literature regarding healthy lifestyle behaviors in children. The SBIRT platform will be developed in partnership between our research team and a third-party intervention development company. Phase II: Focus groups with parents, as well as health care professionals, researchers, and trainees in pediatrics (n=30), will explore intervention-related perceptions and preferences. Qualitative data from the focus groups will inform refinements to the aesthetics, content, structure, and function of the SBIRT. Phase III: Parents (n=200) of children—boys and girls, 5 to 17 years old—will be recruited from a primary care pediatric clinic while they await their children’s clinical appointment. Parents will be randomly assigned to one of five groups—four intervention groups and one control group—as they complete the SBIRT. The randomization function is built into the tool. Parents will complete the eHealth SBIRT using a tablet that will be connected to the Internet. Subsequently, parents will be contacted via email at 1-month follow-up to assess (1) change in concern for, and motivation to, support children’s dietary and physical activity behaviors (primary outcome), and (2) use of online resources and referrals to health services for obesity prevention (secondary outcome).

**Results:**

This research was successfully funded and received ethics approval. Development of the SBIRT started in summer 2012, and we expect all study-related activities to be completed by fall 2016.

**Conclusions:**

The proposed research is timely and applies a novel, technology-based application designed to enhance parents concern for, and motivation to, support children’s healthy lifestyle behaviors and encourage use of online resources and community services for childhood obesity prevention. Overall, this research builds on a foundation of evidence supporting the application of SBIRTs to encourage or “nudge” individuals to make healthy lifestyle choices. Findings from Phase III of this project will directly inform a cluster randomized controlled trial to study the effectiveness of our intervention across multiple primary care-based settings.

**Trial Registration:**

ClinicalTrials.gov NCT02330588; http://clinicaltrials.gov/ct2/show/NCT02330588 (Archived by WebCite at http://www.webcitation.org/6WyUOeRlr).

## Introduction

Childhood obesity is an urgent public health issue. Approximately one-third of Canadian children are overweight or obese [1], a proportion that has doubled over the past 25 years [2]. Pessimistically, the impact of most interventions for managing childhood obesity has been modest to date [3], a point that highlights the need for innovative strategies that are designed to prevent unhealthy weight gain among healthy-weight children (ie, primary prevention) *and* manage excess weight among children with overweight and obesity (ie, secondary prevention). To optimize the effectiveness of such approaches, parents need to play a central role. Specifically, parents set the stage for children’s healthy lifestyle behaviors by fostering a supportive home environment, role-modelling healthy lifestyle habits, and monitoring and reinforcing children’s behaviors [4,5]. Paradoxically, some parents do not perceive their children’s excess weight as a health concern [6], a perception that may be influenced by parents’ inability to accurately recognize obesity in their children [7]. Among parents who have an accurate perception of their child’s weight status (eg, their child meets clinical criteria for obesity *and* parents perceive their child to be obese), only 50 to 60% initiate and sustain healthy lifestyle changes [8]. These results suggest that interventions that attempt to correct parents’ inaccurate perceptions of their children’s weight status *and* enhance their concern for, and motivation to, support children’s healthy lifestyle behaviors may be useful.

Obesity is a common health issue, so interventions to prevent obesity need to be accessible, affordable, and scalable in order to reach a large target audience. The widespread use and availability of the Internet highlights its potential value as a vehicle to deliver obesity prevention interventions [9]—eHealth (electronic health) and mHealth (mobile health) interventions are contemporary terms used to describe health care services and practices that are supported by electronic infrastructure. The benefits of these types of interventions include their ability to offer immediate and tailored feedback, cost-effectiveness, and potential for widespread reach [10]. Web-based interventions may specifically enhance health services by (1) removing social barriers and providing anonymity [11], (2) overcoming limited availability of obesity-related health services [12], and (3) compensating for low confidence and skill levels reported by health care providers [13,14]. Systematic reviews have reinforced such advantages for Web-based interventions for both children [11,15] and parents [16], reporting statistically and clinically meaningful improvements in obesity-related outcomes and lifestyle behaviors.

To date, the majority of online interventions to address obesity-related behaviors have applied time-intensive (eg, online programs up to 52 weeks in length [17]) and resource-intensive (eg, online interventions with additional in-person components [18]) models, suggesting there is value in examining the application of brief and novel online strategies for the prevention of obesity in children [19,20]. One such strategy includes screening, brief intervention, and referral to treatment (SBIRT) approaches, which are time-limited and include an initial screening step followed by the delivery of a short intervention, usually within a 10 to 20 minute period, with options to refer users to treatment and other supportive resources. Fundamental to SBIRTs is the feedback, responsibility, advice, menu options, empathy, and self-efficacy (FRAMES) model [21], which (1) personalizes **f**eedback to communicate unique health outcomes and positive behavior change to the participant, (2) emphasizes personal **r**esponsibility for behavior change, (3) provides **a**dvice on how to initiate and sustain change(s), (4) creates a **m**enu of change options, (5) expresses **e**mpathy, and (6) emphasizes **s**elf-efficacy for change. Historically, SBIRTs have been used to address preventable health concerns (eg, alcoholism, cannabis use) and studies have shown this approach can exert a positive influence on intention to change behaviors as well as behavior change itself [22,23]. SBIRTs are particularly well-suited for obesity prevention in primary care, as providers often have frequent opportunities to interact with families, but limited time and resources to do so. Furthermore, because primary care represents most families’ first point of contact with the health care system [24], the provision of preventative health services, particularly for the primary prevention of chronic diseases, is proactive, efficient, and cost-effective. As well, families tend to access primary care-based health services throughout the life course, so it represents a suitable environment to capture longitudinal data.

With these issues in mind, we hypothesize that an online SBIRT targeting parents will increase their awareness of their children’s weight status and enhance parents’ concern for, and motivation to, support their children’s healthy lifestyle behaviors. The program will have a prevention approach designed to benefit parents with children from across the body weight continuum. Specifically, our SBIRT will encourage parents of children with healthy weights to seek resources to eat healthfully and be physically active to maintain their children’s weight status. It will also guide parents of children with unhealthy weights to access information and health services to improve their children’s weight status and associated health risks. Our three-phase, multi-method study includes the following objectives:

1. Develop an online SBIRT tool designed to raise parents’ awareness of their children’s weight status and lifestyle behaviors.

2. Refine the SBIRT tool by assessing acceptability, satisfaction, and usability using focus groups with pediatric health care professionals, researchers, and parents.

3. Determine the feasibility (pilot-testing) and impact (pragmatic trial) of the intervention through a randomized controlled trial (RCT) design, which will include administering our SBIRT to a sample of parents and collecting data at baseline and 1-month postintervention to assess (1) changes in parents’ concern for, and motivation to, support children’s dietary and physical activity behaviors—primary outcome—and (2) families’ use of resources and health services for the prevention of childhood obesity—secondary outcome.

## Methods

### Study Design

This study includes intervention development (Phase I), refinement (Phase II), and testing (Phase III). Such a design is appropriate when a number of research-related parameters (eg, adverse events, cost-effectiveness, feasibility, power calculation for sample size) remain unknown [25].

### Study Setting

This study is being conducted in the primary care setting and will be performed in the waiting room while parents and children await their upcoming pediatrician appointment. Specifically, we are working in partnership with colleagues who lead the Edmonton Oliver Primary Care Network (PCN), one of more than 40 PCNs in the province. In Alberta, PCNs were developed by our provincial health system to enhance and coordinate health services delivery in primary care. They include family physicians, a multidisciplinary team of health care professionals, decision makers, and administrators, all of whom work collaboratively to address the needs of the local patient population. This setting represents a suitable venue to address the primary and secondary prevention of childhood obesity because (1) PCNs are often families’ first point of contact with the health care system, (2) the goals and priorities of PCNs are aligned with primary and secondary prevention of chronic diseases, and (3) patients typically access health care services at PCNs throughout their lives, which represents an excellent setting to maintain contact with, and collect information from, families over an extended period [24,26].

### Phase I: Development

Our SBIRT tool, which we have titled the Resource Information Program for Parents on Lifestyle and Education (RIPPLE), will be developed in partnership with Evolution Health (EH), a Web-based intervention development company based in Toronto, Ontario [27]. Content in the SBIRT will be incorporated from current literature on children’s healthy lifestyle behaviors, including dietary, physical activity, and sedentary behavior habits, as seen in Slater et al [28] and a review by Steinbeck [29]. Theoretical underpinnings of the SBIRT will be informed by concepts used in existing interventions, for example, the Norm Activation Model [30]. Consistent with the Health Belief Model [31], the SBIRT will be designed to act as a *cue to action*, in which the intervention will prompt parents to initiate and sustain healthy lifestyle changes for their children. Specifically, the SBIRT will act as a trigger by creating a discrepancy between parents’ perceptions of their children’s dietary and physical activity habits and either *normative* or *injunctive* feedback—parents will receive either *normative* feedback on how their child relates to reference norms drawn from the Canadian pediatric population for eating [32] and physical activity [33], or *injunctive* feedback, which includes national recommendations [34]. By providing parents with two types of feedback, we will determine if injunctive or normative feedback is more salient for parents in the context of supporting their children’s healthy lifestyle behaviors.

Based on SBIRTs previously developed by Evolution Health [35,36], our SBIRT will *screen* children’s weight status, which will include sharing this information with parents, as well as deliver a *brief intervention* to parents related to their children’s lifestyle behaviors, and provide *referrals to treatment* and other supportive resources for parents. Specifically, the guided user interface (GUI) within the online program will include the following steps:

1. Data input. Children’s height (cm) and weight (kg) will be measured by the study-designated research assistant (RA) using a wall-mounted electronic stadiometer and an electronic medical scale. The RA will enter this data into the iPad program and then pass the iPad to the parent.

2. Screening. Parents will receive objective, personalized feedback both numerically, based on their children’s body mass index percentile, and visually, using a healthy weight ruler [37].

3. Brief theory-driven intervention. Parents will be randomly assigned to an intervention group, where they will complete one of four brief questionnaire-based interventions, or to the control group, *Heads Up!* The latter includes information on children’s lifestyle behaviors only. Two interventions will include nutrition-based questions—*Eat It!*—and two interventions will include physical activity-based questions—*Move It!* In each of the interventions, parents will receive either *normative* or *injunctive* feedback. Between-group differences in primary and secondary outcomes will be assessed to determine the differential impact of the intervention across the five groups of parents.

4. Toolkit. Parents will be presented with a menu of online resources and community services to choose from.

5. Theory-based measurement. To understand how the SBIRT works to influence parents’ intentions, a brief questionnaire has been adopted from Campbell et al [38] and will assess parents’ concern for, and motivation to, support children’s dietary and physical activity behaviors.

6. Tailored report. Parents will receive a personalized report that will include their children’s weight status, their responses to the intervention questions as well as the feedback they received, and the resources and services they selected from the toolkit*.*

To measure changes in primary (ie, parental concern for, and motivation to, support changes in children’s lifestyle behaviors) and secondary outcomes variables (ie, families’ use of resources and health services), parents will be contacted at 1-month follow-up to complete the same theory-based measurements they completed at baseline and a brief questionnaire to assess their use and/or intention to use the suggested resources and community services. By design, the SBIRT will require parents to indicate their preferred mode of contact for receiving the 1-month follow-up measure and questionnaire (eg, mail, telephone, email), which is designed to optimize participant retention.

### Phase II: Refinement

Following intervention development, focus groups with parents of children aged 5 to 17 years, along with pediatric-focused health care professionals (ie, primary, secondary, and/or tertiary providers with experience in childhood obesity prevention and management), health services administrators, and researchers (ie, faculty and trainees in the field of pediatrics and obesity) will be used to assess acceptability, satisfaction, and usability of the SBIRT tool. Parents will be recruited via word of mouth at the local university where the research is being conducted—a minimum of 10 parents will be recruited in order to gain an adequate representation of the caregiver perspective. Consistent with reports regarding the difficulties in organizing and running focus groups with specific populations [39,40], mechanisms to obtain parents’ perspectives (eg, one-on-one individual interviews) will be used if necessary. Using the recruitment technique of snowball sampling, health care providers, administrators, and researchers (n=20-25) will be purposefully sampled for demographic variation in participant groups (eg, disciplinary orientation, experience). This estimated sample size is consistent with methodological recommendations [41] and similar previous investigations [42,43], which will enable us to attain a high level of data saturation.

In a private room, the RA trained in facilitating focus group interviews will provide eligible participants with a study explanation and formal invitation to participate—informed, written consent will be obtained. Focus groups—6 to 8 participants per group—will occur over four to five sessions, and be 60 to 90 minutes in duration. The RA will lead groups step by step through the SBIRT (version 1.0) by entering standardized data into the GUI and projecting the Web-based content onto a screen. Interviews will include open-ended questions to query (1) participants’ overall impressions of the intervention, (2) factors related to participants’ acceptability of, and satisfaction with, the intervention, and (3) theoretical underpinnings of the SBIRT. Probes will include prompts on SBIRT perceptions, preferences, and how to best incorporate the program into existing clinical processes—perceived strengths, limitations, and areas for improvement will also be explored. A closing discussion will query perceptions of the need for long-term support within and beyond primary care and how this support should be provided.

Focus group discussions will be transcribed in real time using a court reporter, which optimizes transcription accuracy and ensures confidentiality [44]. Data will be managed and analyzed using NVivo 10 (QSR, Melbourne, Australia). Qualitative data analysis is a cognitive process that includes comprehending, synthesizing, theorizing, and recontextualizing [45], and the method of qualitative description [46] will be used to develop a basic description of the data. Data will be analyzed in a line-by-line process. From the initial analysis, a coding scheme will be developed to identify all meaningful units and new themes will be added as necessary. Once each discussion is coded, themes will be grouped under general categories and a written description will be constructed to explain each category. To enhance methodological rigor, we will (1) triangulate participants’ views by interviewing health care professionals, researchers, and parents, (2) employ concurrent data collection and analysis to inform amendments to the interview guide, and (3) implement a real-time member-checking protocol to ensure findings accurately reflect participants’ personal perspectives—at the end of each group, the moderator will confirm and clarify discussed themes [39].

### Phase III

#### Pilot-Testing

The objective of this phase is to pilot-test the refined SBIRT with parents (n≈30) to determine the feasibility of incorporating the intervention in primary care, including (1) accuracy of the randomization procedures, (2) ability to retain participants at follow-up, (3) practicality of clinician involvement, (4) suitability of the primary and secondary outcome measures, and (5) time to complete the intervention in the primary care waiting room. All of these elements are important to assess prior to determining the effectiveness of a newly developed intervention [25,47]. Upon recruitment and 1-month follow-up, the researchers will cease participant recruitment for a 2-week period to assess issues regarding feasibility—at this time, modifications may be made to study processes and procedures before initiating the pragmatic trial.

#### Pragmatic Trial

##### Trial Design

A parallel-group, double-blinded randomized controlled trial will be used to assess the effectiveness of the intervention. The trial has been registered publically (ClinicalTrials.gov identification number: NCT02330588) [48] and adheres to CONSORT guidelines [49]. Participants will be recruited and enrolled by an RA and RIPPLE will assign a unique, nonidentifying number to participants. The allocation sequence will be electronically generated within the GUI and to reduce the risk of selection bias, participants (n=200 parents) will be randomly assigned to one of five groups—*Eat It!* (normative), *Eat It!* (injunctive), *Move It!* (normative), *Move It!* (injunctive), or *Heads Up!* (control group). Group assignment will be done using blocked randomization—five arms, block size of five—to ensure equal group sizes—n=40/arm, equal allocation ratio of 1:1—throughout the study. To reduce the risk of performance bias, the RCT will be double-blinded. Specifically, the study-designated RA (JLSA) will not be aware of participants’ intervention assignment, unless participants request assistance with the program, thus potentially revealing their assignment. Research personnel will be blinded for the remainder of the research process, including outcome assessment, in order to minimize the risk of detection bias [50]. As well, study participants will not be aware of their assignment to the intervention or control groups. Prior to the intervention, participants will receive information that is sufficient to obtain informed consent, but inadequate so as to decipher between intervention groups. Although contamination is a possibility given the close proximity of participants, given that only one participant can be recruited at a time, enrollment and recruitment will be staggered and the opportunity for participants to discuss the intervention with each other is unlikely.

##### Sampling and Recruitment

Parents of children awaiting their pediatrician appointment will be recruited for the RCT. During this time, the RA will liaise with the intake nurse to identify families who are suitable for study participation. Families will be eligible for study inclusion if (1) children present with nonurgent medical issues, (2) children are 5 to 17 years of age, and (3) children attend their appointment with at least one parent. Parents (eg, mothers, fathers, legal guardians) will be eligible if they (1) self-identify as a child’s primary caregiver, and (2) speak and read English. The nurse will also help to differentiate urgent (eg, febrile, acute asthma attack) and nonurgent (eg, medical checkup, asthma follow-up) presentations, of which only the latter will be approached for recruitment. Families who are identified as eligible by the clinic nurse will be approached by the study RA in the waiting room of the primary care clinic. Average wait times in the Edmonton Oliver PCN are 15 to 30 minutes, so this time will be used to (1) recruit participants, (2) obtain informed, written consent (adult) and assent (child), (3) measure and input children’s anthropometric data, and (4) deliver the brief, online intervention to parents on the study-designated tablet (iPad). If families present with more than one child and both are interested in participating, only the child with the next upcoming birthday will be enrolled—this will be done to ensure that each study participant represents an independent case. As a token of appreciation and to encourage parents to complete the 1-month follow-up measure, parents will be given a Can $25 gift card to a local business (eg, grocery store).

**Figure 1:**
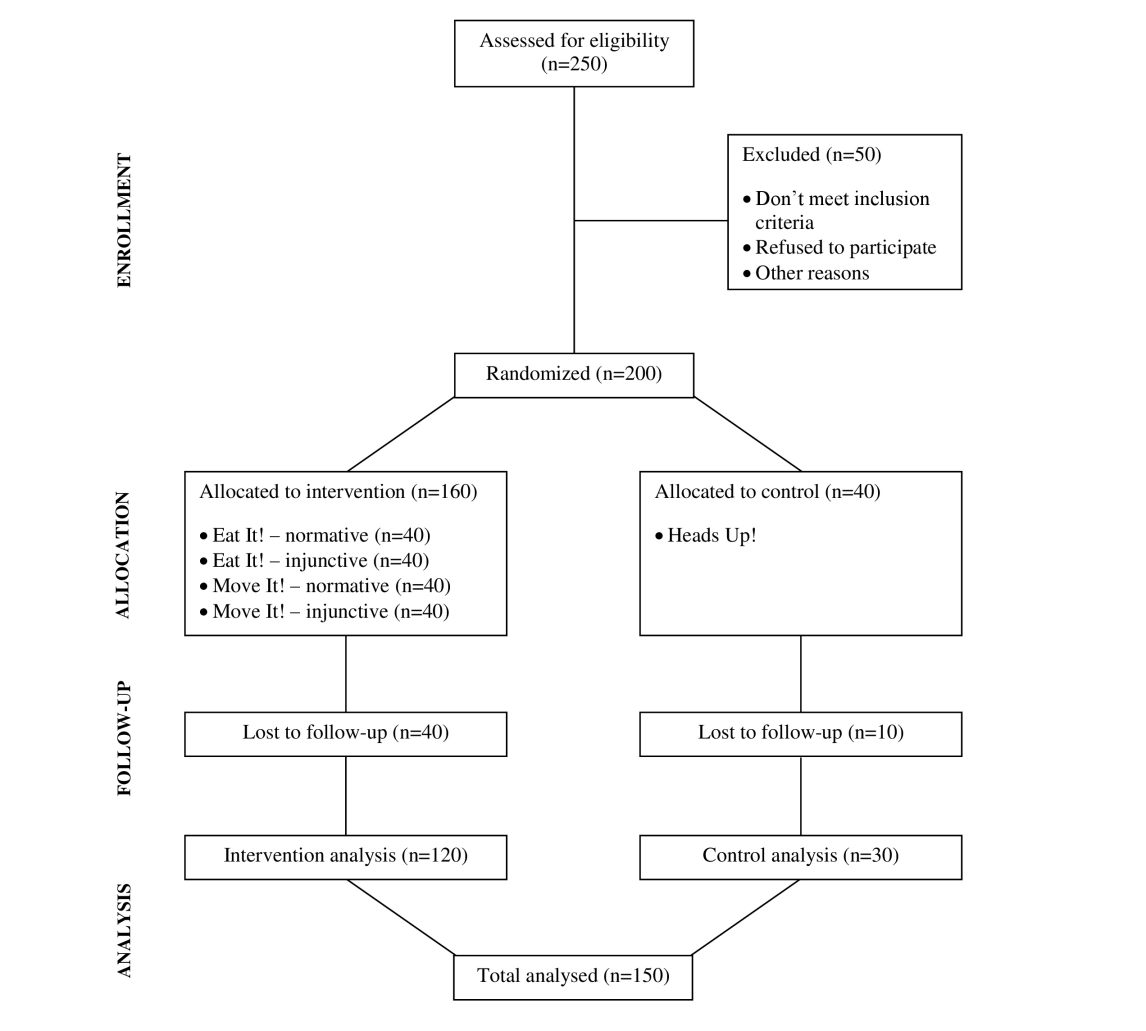
Flow diagram of predicted progress through RCT phases (enrollment, allocation, follow-up, and analysis).

Primary health care providers at this site have historically had around 2500 patient encounters per year [51], so our research staff will recruit families on approximately 2 days per week over several months to accumulate our study sample. Given the design of the SBIRT, parents will also need adequate time to complete everything at once (ie, saving and completing the intervention at a later date will not be possible within the SBIRT). With time constraints in mind, families will be approached to participate in the study if we—clinical/administrative staff, research team—believe parents will have sufficient time (ie, 15 to 20 minutes) to complete the intervention and research procedures while waiting for their scheduled clinical appointment. Although it is a potential barrier that some recruited parents may be unable to complete the program while they wait, previous studies have supported the feasibility of brief online interventions under similar conditions, for example, Freeborn et al [52]. Assuming 20 to 30% attrition at 1-month follow-up [53], complete data from approximately 150 parents is expected. Figure 1 represents a flow diagram of expected recruitment and retention. Based on primary care client demography, this sample size will allow us to enroll a diverse group of families that vary by age, ethnicity, family income, and weight status.

##### Data Collection

Within the GUI, we will collect the following information (see Case Report Form in Multimedia Appendix 1): families’ demographic information, children’s weight status, resources and services chosen by parents, and parents’ responses to both the intervention questions (unless allocated to the control group) and the theory-based questionnaire (see Phase I in Methods).

##### Data Management and Analysis

Security measures that adhere to provincial and federal privacy requirements will be integrated into the program. Access to the online SBIRT will be password protected, all data transactions between the Web and data services will be encrypted, and the server will be located behind a firewall to safeguard personal data.

Quantitative data analyses will be performed using SPSS version 22.0 (SPSS Inc, Chicago, IL, USA). Continuous variables will be described by univariate summaries, and frequency distributions will be determined for categorical variables. Box plots and histograms will display continuous variables, and bar charts will display categorical variables. Recruitment rate and participant characteristics (eg, sex, weight status, demographics) will be calculated to assess enrolment tendencies and biases in subgroups. Retention rate, or the proportion of participants who remain in the study at 1-month follow-up, will be calculated to assess the likelihood of attrition within and between subgroups. A bivariate statistical model (eg, Wilcoxon rank-sum test) will be used to compare program completers versus noncompleters to assess attrition bias. Hierarchical linear modeling [54] will be used to assess intra- and interlevel individual differences and group changes in the predetermined outcomes. Specifically, the primary and secondary outcomes will be assessed at the individual level (ie, nested within each intervention group) and group level (ie, between each intervention group), both at baseline and at 1-month follow-up. This form of analysis is appropriate when observations are nested within groups and/or multiple time points. Statistical significance will be set at *P*<.05.

## Results

This is a three-phase, multi-method study designed to build, refine, and complete testing of an online SBIRT to enhance parents’ concern for, and motivation to, support children’s healthy lifestyle behaviors. Development of the project commenced in summer 2012, and the expected date of completion is fall 2016 (Table 1). The Health Research Ethics Board at the University of Alberta (Edmonton, AB) has approved this study.

**Table 1:** Timeline of study-related activities.

Study activities and substeps		Year and season^a^of study															
		2012		2013				2014				2015				2016			
		S	F	W	Sp	S	F	W	Sp	S	F	W	Sp	S	F	W	Sp	S	F
**Preparatory activities**																	
	Organization of the research team	✓																	
	Ethics application		✓																
	Develop the toolbox of resources		✓	✓															
**Study activities**																	
	Phase I: Development			✓	✓	✓	✓	✓	✓	✓									
	Phase II: Refinement										✓	✓							
	Phase III: Testing												✓	✓	✓				
**Knowledge translation**																	
	Formal meetings		✓		✓		✓		✓		✓		✓		✓		✓		
	Research blog updates						✓	✓	✓	✓	✓	✓	✓	✓	✓	✓	✓	✓	✓
	Findings dissemination												✓					✓	✓

^a^Summer (S), Fall (F), Winter (W), Spring (Sp).

## Discussion

### Distinctive Features

This paper highlights the study protocol for the development, refinement, and testing of a novel SBIRT designed to enhance parents’ concern for, and motivation to, support children’s healthy lifestyle behaviors, as well as link them with relevant resources and services to help prevent childhood obesity in primary care. Historically, SBIRTs have been developed and applied to facilitate positive changes related to addictive behaviors (eg, cannabis use, problem drinking). A recent review and meta-analysis of SBIRTs for screening of alcohol consumption in primary care found that the majority of participants across 22 trials demonstrated positive behavior change (ie, reduced consumption) at 12-month follow-up [23], a finding that suggests the positive effects of this brief approach may have the potential for longevity. Furthermore, in comparison to lengthier online interventions (ie, 60 minutes or longer), positive outcomes of brief interventions were not statistically different [23], highlighting that the dosage of exposure is not necessarily proportionate to the treatment effect, thus justifying the use of a time- and resource-limited approach. In addition to the potential for positive behavior change, specific program elements unique to SBIRTs (eg, automatic screening procedures, personalized feedback, menu of resources and services) are well-suited for primary care. Although this setting prioritizes frontline prevention of chronic diseases, primary care providers report a number of barriers and challenges to fulfilling this task [13,55]. Our intervention may address this deficit and reduce the pressures and expectations faced by primary care health care providers who often lack confidence and skills in preventing childhood obesity [14]. Our SBIRT may also help families to overcome limited availability of health services for the secondary prevention of childhood obesity (eg, multidisciplinary weight management clinics, outpatient nutrition counseling). Lastly, our Web-based SBIRT for parents may enhance existing resources and health services for obesity prevention in children. For instance, the intervention may remove social barriers and provide anonymity for families that are reluctant to receive care and support in person.

Although the application of SBIRTs to the prevention of obesity in children remains untested, recent systematic reviews [11,15] have highlighted the advantages associated with online interventions (eg, family-based Internet programs, Internet counselling, Web-based interactive behavior programs) that are aligned with RIPPLE with respect to the aim of obesity prevention in children. For example, an online primary care-based program for preventing childhood obesity was well-received by clinicians and families, in which clinicians were more likely to speak with families about healthy weights, and parents intended to increase their children’s vegetable and fruit intake, postintervention [26]. It is noteworthy, however, that the majority of such interventions have focused on time- and resource-intensive models, approaches that are often difficult to implement and sustain, particularly in primary care. Additionally, given that online approaches represent a relatively new niche of study, little is known regarding the impact of online interventions on weight-related health outcomes and intentions to change lifestyle behaviors [56]. Taken together, there is a real need to develop and evaluate brief, Web-based interventions to help connect families with relevant resources and services that may *nudge* them towards healthy behavior changes in a setting where parents and children are already present and waiting.

### Study Strengths

This project was developed in direct response to health systems gaps and priority areas in Canada. To date, the research team has received strong support from health care professionals and provincial health care organization decision makers, highlighting the support for, and relevance of, our research. Second, this research will directly inform how such a brief, parent-based approach to address childhood obesity can be incorporated into everyday clinical practice in primary care. Providing families with tailored feedback, practical resources, and information on local health services will help to overcome clinical barriers associated with the primary and secondary prevention of obesity in children. Lastly, findings from this developmental study will inform a future cluster clinical trial to test effectiveness of the intervention across multiple PCN clinics. Specifically, results from Phase III of this study will (1) help to estimate preliminary effect sizes of the SBIRT, informing a sample size calculation for a future cluster RCT, (2) confirm our ability to recruit and retain participants from primary care, and (3) determine appropriateness of primary and secondary outcomes, and follow-up time points.

### Study Limitations

We acknowledge that our SBIRT is new and remains untested. Given this reality, there are a number of program components (eg, theoretical underpinnings, duration of follow-up time period) that have been informed by related projects [57,58]. Because a number of research-related parameters remain unknown, our study design includes modifying the intervention (Phase II) prior to formal testing (Phase III), which will facilitate refinement of program structure, function, language, and aesthetics before initiating testing with parents in Phase III. We also appreciate that most SBIRTs have investigated participants’ motivation to change their own individual behaviors, whereas our study assesses the motivation of parents to help change their children’s behaviors, in other words, surrogate motivation. Given this degree of separation, parents’ motivation may neither accurately reflect children’s motivation to make lifestyle behavior changes, nor be sufficient to initiate behavior change. These are relevant issues that will be explored further in follow-up research that will build on this initial study.

### Conclusions and Future Directions

Our applied health services research is timely and the objectives align with research priorities to prevent obesity in children. This protocol study encompasses the development, refinement, and testing of a parent-based, online SBIRT that will directly inform the feasibility of incorporating such an approach into everyday clinical practice. By providing families with tailored feedback and information on applicable resources and community services, our SBIRT will encourage family self-management of obesity-related behaviors in primary care. Findings from this project will confirm a number of feasibility-related parameters in the pilot study (eg, feasibility of incorporation into primary care, recruitment and retention rate, suitability of primary and secondary outcome measures) and preliminary effectiveness of the intervention, which will be tested in a future cluster RCT.

## Multimedia Appendix 1

Case Report Form.

## Multimedia Appendix 2

CIHR Funding Reviews.

## Multimedia Appendix 3

CIHR Funding Information.

## Abbreviations

AI-HS: Alberta Innovates – Health Solutions
CIHR: Canadian Institutes of Health Research
EH: Evolution Health
FRAMES: feedback, responsibility, advice, menu options, empathy, and self-efficacy
GUI: guided user interface
PCN: Primary Care Network
RA: research assistant
RCT: randomized controlled trial
RIPPLE: Resource Information Program for Parents on Lifestyle and Education
SBIRT: screening, brief intervention, and referral to treatment
