# Supplementary material for: Working With Parents to Prevent Childhood Obesity: Protocol for a Primary Care-Based eHealth Study
Source: JMIR Res Protoc. 2015 Mar 25;4(1):e35. doi: 10.2196/resprot.4147 (PMC4390613; doi:10.2196/resprot.4147)
Supplement: Supplementary file 1 [file resprot_v4i1e35_app1.pdf]

CASE REPORT FORM

Question	Specific Details of Data Entry
**Researcher Entry**	
1. Enter the child's measured height.	To the nearest 0.00cm
2. Enter the child's measured weight.	To the nearest 0.00kg
**Parent Entry: Pre-Intervention**	
1. Please enter your contact information:	
a. Primary Phone Number	xxx-xxx-xxxx
b. Secondary Phone Number (optional)	xxx-xxx-xxxx
c. Email	
d. Parent's First Name	
e. Child's First Name	
2. What is your child's sex?	Male/Female
3. What is your child's date of birth?	mm/dd/yyyy
4. What is your relationship to the child?	Biological mother, Biological father, Step-mother, Step-father, Adoptive mother, Adoptive father, Foster mother, Foster father, Grandmother, Grandfather, Sister, Brother, Aunt, Uncle, Cousin, Legal guardian, Prefer not to say
5. Are you the child's primary caregiver?	Yes/No
6. What is your ethnic background?	White (*e.g.*, Northern European), Aboriginal (*e.g.*, Metis), Asian (*e.g.*, Chinese), Black (*e.g.*, African American), Latino (*e.g.*, Mexican), Southeast Asian (*e.g.*, East Indian), Mixed, Other
7. What is your child's ethnic background?	White (*e.g.*, Northern European), Aboriginal (*e.g.*, Metis), Asian (*e.g.*, Chinese), Black (*e.g.*, African American), Latino (*e.g.*, Mexican), Southeast Asian (*e.g.*, East Indian), Mixed, Other
8. What is your total household income?	$0 – 20 000, $20 001 - $40 000, $40 001 - $60 000, $60 001 - $80 000, $80 001 - $100 000, over $100 000, Prefer not to say, Don't know
9. What is your highest level of education?	Some high school, Completed high school, Some college/university, Completed college/university, Graduate degree, Prefer not to say
10. What is your current relationship status?	Single, Common Law, Married, Divorced, Separated
11. How would you describe your child's weight?	Very underweight, A little underweight, Average, A little overweight, Very overweight
**Parent Entry – Intervention**[*]	
**Parent Entry: Post-Intervention for *Eat It!***[†]	
1. How concerned are you about your child's weight or body size?	1 – 5 (1:Not Concerned; 5:Very Concerned)
2. How important is it to you, right now, to change what your child eats?	1 – 5 (1:Not Important; 5:Very Important)

3. How much, at this moment, do you personally want to change what your child eats?	1 – 5 (1:Not At All; 5:Very Much)
4. How confident do you feel about succeeding in changing your child's eating habits?	1 – 5 (1:Not Confident; 5:Very Confident)
5. How confident do you feel about succeeding in having your child eliminate regular soda, fruit, juice, or sports drinks?	1 – 5 (1:Not Confident; 5:Very Confident)
6. How ready are you to change your child's eating habits?	1 – 5 (1:Not Ready; 5:Very Ready)
7. I intend to discuss my child's weight with our doctor today.	1 – 5 (1:Strongly Disagree; 5:Strongly Agree)
8. During the next month, I intend to use the resources and/or services that I selected in the previous section of this program for my child.	1 – 5 (1:Strongly Disagree; 5:Strongly Agree)

Parent Entry: Post-Intervention for *Move It!*[†]

1. How concerned are you about your child's weight or body size?	1 – 5 (1:Not Concerned; 5:Very Concerned)
2. How important is it to you, right now, to have your child engage in a more physically active lifestyle?	1 – 5 (1:Not Important; 5:Very Important)
3. How much, at this moment, do you personally want to change your child's level of physical activity?	1 – 5 (1:Not At All; 5:Very Much)
4. How confident do you feel about succeeding in changing your child's level of physical activity?	1 – 5 (1:Not Confident; 5:Very Confident)
5. How confident are you that you can engage your child in one hour of moderate-to-vigorous physical activity each day?	1 – 5 (1:Not Confident; 5:Very Confident)
6. How ready are you to change your child's level of physical activity?	1 – 5 (1:Not Ready; 5:Very Ready)
7. I intend to discuss my child's weight with our doctor today.	1 – 5 (1:Strongly Disagree; 5:Strongly Agree)
8. During the next month, I intend to use the resources and/or services that I selected in the previous section of this program for my child.	1 – 5 (1:Strongly Disagree; 5:Strongly Agree)

Parent Entry: Post-Intervention for *Heads Up!*[Δ]

---

[*]In development.
[†]Questions have been adopted, with permission, from Campbell et al. (2011) [38].
[Δ]Includes questions from the Post-Intervention for *Eat It!* (1 – 6) and *Move It!* (2 – 8).
