# Supplementary material for: Working With Parents to Prevent Childhood Obesity: Protocol for a Primary Care-Based eHealth Study
Source: JMIR Res Protoc. 2015 Mar 25;4(1):e35. doi: 10.2196/resprot.4147 (PMC4390613; doi:10.2196/resprot.4147)
Supplement: Supplementary file 2 [file resprot_v4i1e35_app2.pdf]

**Application Number / Numéro de demande:**	267442
**Name of Applicant / Nom du chercheur:**	BALL, Geoff D.C.
**Review Type / Type d'évaluation:**	Committee Member 1/Membre de comité 1
**Competition:**	2011-11-01 Partnerships for Health System Improvement (PHSI)
**Concours:**	2011-11-01 Partenariats pour l'amélioration du système de santé (PASS)
**Committee:**	Partnerships for Health System Improvement
**Comité:**	Partenariats pour l'amélioration du système de santé

Potential Impact

Comments:

The specific aim of this proposal is to develop and test a web-based, universal “screening and brief intervention” (SBI) protocol designed to motivate parents to (1) support healthy eating and physical activity in their children and (2) access community resources and health services to prevent childhood obesity. This study will be conducted in partnership with the Edmonton Oliver Primary Care Network (EOPCN) and include 3 phases: 1) to develop the SBI protocol designed to motivate parents to eat healthfully and be physically active to maintain their weight status (1ry prevention) or reduce obesity and associated health risks (2ry prevention); 2) to refine the SBI protocol by assessing usability, acceptability, viability, and satisfaction using focus group interviews with parents and EOPCN clinicians; 3) to gather data with 140 families at baseline, 1-, and 6-month follow-up on recruitment & retention rates, body weight distribution of enrolled children, and preliminary evaluation of effect sizes for the primary objectives in order to plan for a future RCT. If successful, this research proposal will encourage self-management by providing families with tailored feedback and linking them with appropriate information and health services, and will also generate preliminary data and acquire valuable team experience to inform a future RCT to examine the efficacy of this web-based SBI for parents to prevent childhood obesity.

The study is highly relevant to the Canadian health system since childhood obesity is common and increasing in prevalence, and usually translates in adult obesity and related complications. Targeting childhood obesity and particularly families is important in order to counteract the global epidemic of obesity in Canada. The fight against obesity is a priority for provincial health services throughout Canada.

The research team is well implicated in initiatives to improve health services and evaluation of obesity care in Alberta. Relevant decision makers are implicated in the proposal and have already collaborated with the research team on other obesity-related initiatives.

Results of the proposed research will probably not have the appropriate level of evidence (no control group) to definitively impact policies and practices at this point. They will mainly serve to plan a future RCT, which is an appropriate outcome at this stage of development of the proposed tool.

If outcomes of this study are conclusive, it is unclear how the proposed web-based tool is planned to be used in the primary care setting in the future RCT or in practice. For this study, 140 families will be selected in EOPCN waiting rooms and will access the tool using a study-dedicated iPad. It is mentioned that the logistical issue of parents’ Internet access to complete follow-up procedures will be clarified at baseline to determine how parents will access our SBI. So in the future, when the SBI will have to be implemented in primary care clinics: 1) Does the team plan that families will be provided with access to the SBI at the primary care location using an iPad? 2) If not, how will the families be informed of the web-based tool and invited to log-on? If yes, who will provide this access in the clinic and how? 3) Is it expected that most families at risk for childhood obesity have access to the internet and knowledgeable for its use, considering that prevalence of childhood obesity is increased in low-income families? These issues for the future implementation of their tool should have been discussed in the proposal.

**Application Number / Numéro de demande:**	267442
**Name of Applicant / Nom du chercheur:**	BALL, Geoff D.C.
**Review Type / Type d'évaluation:**	Committee Member 1/Membre de comité 1
**Competition:**	2011-11-01 Partnerships for Health System Improvement (PHSI)
**Concours:**	2011-11-01 Partenariats pour l'amélioration du système de santé (PASS)
**Committee:**	Partnerships for Health System Improvement
**Comité:**	Partenariats pour l'amélioration du système de santé

Scientific Merit

Comments:

The research questions are clearly related to the funding opportunity and have been identified by the decision makers of the research team as relevant and timely.

The proposed mixed methods are well thought through and appropriate, and will enable the research questions to be answered. Main potential limitations are discussed. A major advantage is having a very talented team which includes many relevant expertise and many relevant stakeholders bought into the project from the outset. The KT plan is robust and diversified, and the team is experienced in KT activities.

The number of families who will be approached for the 1st objective should be stated. How will this objective be evaluated? There is no mention of feedback or satisfaction assessment at this phase in order to achieve the proposed phase 1 outcome, i.e. “a draft version of our SBI to undergo refinement”. At the 3rd step of the SBI tool, it is mentioned that a brief intervention is provided. But this is not explained. The proposal just mentions that questions “will let us examine how parents perceive and facilitate their children’s obesity-related behaviours”.
Regarding the 3rd phase of the project, no control groups will be used to compare the results of the intervention. This phase is a essentially a pilot study in order to plan a future RCT.

Track record in related areas based on previous funding and publications is excellent. The team gathers all required expertise to complete successfully the proposal and generate relevant data. The project is accomplishable in 3 years with the resources described. But the proposal does not comment on the feasibility to recruit 140 families within the proposed timeframe. As mentioned, the findings of this study, when available, will not be applied to change policies or practice, but to support and plan a future RCT. It is also unclear how the tool will be implemented in real clinical practice in the future.

The budget is appropriate.

**Application Number / Numéro de demande:**	267442
**Name of Applicant / Nom du chercheur:**	BALL, Geoff D.C.
**Review Type / Type d'évaluation:**	Committee Member 2/Membre de comité 2
**Competition:**	2011-11-01 Partnerships for Health System Improvement (PHSI)
**Concours:**	2011-11-01 Partenariats pour l'amélioration du système de santé (PASS)
**Committee:**	Partnerships for Health System Improvement
**Comité:**	Partenariats pour l'amélioration du système de santé

Potential Impact

Comments:

Purpose:
The project is a well thought out approach to the development of an understanding of Childhood Obesity, acknowledged to be an urgent Public Health issue. The emphasis on primary-care based services for childhood obesity aligns directly with the provincial Obesity Strategy and targets a service gap in primary care. Implications are cross jurisdictional, and findings will inform health care delivery practice and approach.

This project will address primary care clinician barriers to providing effective obesity-related health services and encourage self-management by parents /families . The research team will develop a web based program for parents which will provide personalized feedback for families and support parents of healthy weight children in encouraging healthy eating and physical activity; and guide parents of unhealthy children to access community information and services to reduce obesity and health risks.

This research will directly inform how primary care can provide a brief, parent-based approach to address childhood obesity that can be incorporated into everyday clinical practice. Providing families with tailored feedback, practical educational tools, and information on local health services addresses clinical barriers and encourages self-management of obesity-related behaviours.

Approach:
The research team will partner with the Edmonton Oliver Primary Care Network (EOPCN) - an organization with chronic disease management, obesity, and pediatrics as priorities.

This project has been segmented into three phases:
-Phase one sees the development of a web based program for parents
-Phase two will assess the acceptability, usability and viability of the program using focus group interventions of both parents and health professionals
-Phase three will focus on data collection to determine the feasibility of this approach (determine likelihood of enrollment); Phase three will also collect data to assess preliminary impact of the SBI (by measuring changes in parental concern for and motivation to support child's dietary and physical activity behaviours).

Participants will be recruited through the Edmonton Oliver Primary Care Network through distributed posters and brochures, and by direct approach of RA's. Preliminary eligibility will be determined and informed consent obtained.

The Clinical Research Informatics Core , the Women ad Childrens Health Research Institue and the U of Alberta will assist with export of data into OpenClinicia (OC) database. A rigorous data management program is identified.

Quantitative data analysis will be led by a supervised RA using Stata 11. Descriptive analyses will be completed and recruitment data calculated. Exploratory analyses will include both cross-sectional and longitudinal analyses of changes in the primary outcome variables (parental concern and motivation;

Potential Impact

Comments:

resource and health services utilization).

Interviews will include open-ended questions to query micro- (parent),meso- (family), and macro-level (clinic, health system) factors. Study RA will complete training in qualitative research methods with Dr. Holt (Co-I).

Data on these primary outcomes (concern and motivation; use of resources and health services) will result in an estimation of preliminary effect sizes of the SBI, informing a sample size calculation for a future RCT.

Originality:
The proposed applied health services research is very timely. Objectives align with provincial and national research priorities to prevent childhood obesity as well as plans for comprehensive and integrated obesity care in Alberta. As stated, no research yet explores the utility of a web-based, universal screening and brief intervention (SBI) protocol designed to help parents prevent childhood obesity through the primary care setting.

Applicants:
Applicants demonstrate a depth and breadth of knowledge, are acknowledged leaders in the national leaders in obesity research, are lined and integrated with decision makers and demonstrate a well-established record of KT in related research endeavors. The team consists of both provincial and national leaders who have been involved in initiatives to improve health services and evaluation of obesity care. The team is comprised of key decision makers and researchers who have worked together in research-related capacity previously, and all have extensive experience in both community and intervention research. The role of decision makers is to ensure research relevance within the provincial health service context.

Decision-makers have played crucial roles in developing this proposal and have committed to maintain high levels of participation throughout the study.

There is a direct link to AHS-based decision-makers who developed the provincial obesity plan with the front-line, interdisciplinary clinicians charged with its implementation.

Environment/Impact /Knowledge Transfer:

Potential Impact

Comments:

Given the high prevalence of childhood obesity, there is an urgent need for innovative, scalable approaches that can be delivered in primary care.

Donaldson-Kelly co-leads an interdisciplinary (family medicine, pediatrics, health promotion, primary care, nutrition services, home care) group of stakeholders on Edmonton's Zone Integration Plan (ZIP) committee, tasked with implementing the AHS Obesity Strategy over the next five years. Her leadership and connections with the other four regional ZIP committees (Calgary, North, Central, South) will ensure that knowledge from our research is shared throughout this established organizational network.

There is an integrated KT plan that links and aligns team including in-person team meetings annually, quarterly teleconferences, and informal meetings.

End of KT activities include an incredibly strong (and impressive) set of evidence based strategies including:
•Two graduate trainees (RAs) will present study data at an international meeting (The Obesity Society) and interim data at local (WCHRI Research Day) and national (Accelerating Primary Care) conferences.

•Three peer-reviewed publications from this research.
•Study protocol will be submitted to an open-access journal (BMC Health Services Research); manuscripts based on our quantitative and qualitative data will be submitted to Preventive Medicine and Obesity, respectively.
•An authorship agreement for presentations, abstracts, and manuscripts will be developed collaboratively
•CIHR funding will be acknowledged in all KT activities.
•Peer-reviewed publications will be submitted to PubMedCentral Canada - a free digital archive of peer-reviewed health science research.
•Final Report, Policy Brief, Newsletter will be used to reach audiences: individuals interested in childhood obesity and families dealing with obesity-related issues.
•A final report of our findings will follow a formula developed by the CHSRF. Written by Dr. Ball and co-authored by all team members, this plain-language report will communicate main findings and implications, with national, provincial, and local reach. We will publish it on the Canadian Obesity Network (CON) web site in the pediatric domain (www.obesitynetwork.ca), co-led by Dr. Ball.
•Study-related findings will be disseminated as' promising practices' in AHS publications.
•A policy brief, with recommendations for incorporating childhood obesity-related health services in primary care, will accompany the final report to key policymaking associations e.g., the Canadian Paediatric Society, the College of Family Physicians of Canada, Dietitians of Canada, and the Canadian

Potential Impact

Comments:

Nurses Association.
•A newsletter with a summary of findings will be sent to all participating families and posted on the CON website.
•An article for the two monthly newsletters of the Primary Care Initiative (www.albertapci.ca) that circulate to >40 PCNs throughout Alberta will be produced.
•Public affairs offices from AHS and the UA will interpret findings for local and national media outlets.

Budget:
No issues.

Scientific Merit

Comments:

This project is also methodologically strong.

This proposed mixed methods (qualitative and quantitative) study is a three-phase demonstration project. Knowledge gained from this formative research will guide the development of a future RCT to evaluate (i) intervention efficacy, (ii) processes, time course, and magnitude of behavior change, and (iii) cost-effectiveness. Given this, the project is designed to ensure viability of future RCT success and this enhances the quality of the study, which is particularly important given the need to improve methodological rigour in pediatric research. The study will be registered publically (www.clinicaltrials.gov) and adhere to CONSORT reporting guidelines.

Quantitative data analysis will be led by a supervised RA using Stata 11. Descriptive analyses will be completed and recruitment data calculated. Exploratory analyses will include both cross-sectional and longitudinal analyses of changes in the primary outcome variables (parental concern and motivation; resource and health services utilization). Estimates of effect sizes will be based on percent change in the primary outcomes. Changes across sub-groups will be tested using chi-square tests, two-way ANOVA or the Tukey-Kramer test for post hoc comparisons for unequal sample sizes. Significance will be set at $p<0.05$.

Digitally-recorded interview data will be transferred electronically to the Comma Police (see attached collaboration letter) for transcription, and will be managed and analyzed using N-VIVO 8 (QSR, Melbourne, Australia). Qualitative data analysis is a cognitive process that includes comprehending, synthesizing, theorizing, and re-contextualizing. Analysis will be led by an RA, under the supervision of Dr. Holt, using Morse and Field’s approach.
