# Supplementary material for: Working With Parents to Prevent Childhood Obesity: Protocol for a Primary Care-Based eHealth Study
Source: JMIR Res Protoc. 2015 Mar 25;4(1):e35. doi: 10.2196/resprot.4147 (PMC4390613; doi:10.2196/resprot.4147)
Supplement: Supplementary file 3 [file resprot_v4i1e35_app3.pdf]

April/Avril 30, 2012

DC0190GP

Canadian Institutes of Health Research / Instituts de recherche en santé du Canada

Notice of Decision / Avis de décision

Application Number/Numéro de la demande: 267442

Committee Code/Code du comité: PHE

Applicants/Candidats: Dr. Geoff D.C. BALL   Ms. Angela ESTEY

With/Avec:				
	Dr. A. CAVE	Ms. S. DONALDSON KELLY	Ms. C. ELLENDT	Dr. N. HOLT
	Dr. S. JELINSKI	Ms. P. MARTZ	Dr. K. MAXIMOVA	Dr. R. PADWAL
	Dr. C. WILD			

Institution paid/ Établissement payé: University of Alberta

Title/Titre: Working with Parents to Prevent Childhood Obesity: A Primary Care-based Study

Primary Inst./ Inst. principal: Human Development, Child and Youth Health

Other Related Inst./ Autres inst. connexes: Nutrition, Metabolism and Diabetes; Health Services and Policy Research

**Competition Outcome/Résultats du concours:**	Partnerships for Health System Improvement (PHSI)<br>November/Novembre 01, 2011
**Number in competition/Nbre de demandes dans le concours:**	61
**Number approved/Nbre de demandes approuvées:**	27

**Decision on your application/ Décision sur votre demande:**	Approved
**Average annual amount/ Montant annuel moyen:**	$115,300
**Equipment amount/ Montant pour les appareils:**	$4,100
**Term/Durée:**	**3 yrs/ans 0 months/mois**

Peer Review Committee Recommendation, for your information and use/ Recommandation du comité d'examen par les pairs, pour fins d'information et d'utilisation:

**Committee/Comité:**	Partnerships for Health System Improvement
**Application rank within the competition/ Rang de la demande dans ce concours:**	18
**Percent Rank Within the Competition/ Rang en pourcentage au sein du concours:**	29.51%
**Rating/ Cote:** Potential Impact	3.83
Scientific Merit	3.93
**Recommended average annual amount/ Montant annuel moyen recommandé:**	$115,300
**Recommended equipment amount/ Montant recommandé pour les appareils:**	$4,100

*** Applications receiving a score of less than 3.5 on any evaluation criteria will not be considered for Funding. / Les demandes qui ont reçu une note inférieure à 3.5 pour n'importe quel des critères d'évaluation ne sont pas admissibles.

This document is for information only. Official payment is stated on the CIHR Authorization for Funding.
Document à titre d'information seulement. Le paiement officiel est indiqué sur l'autorisation de financement des IRSC.
